# Fast growth associated with aberrant vasculature and hypoxia in fibroblast growth factor 8b (FGF8b) over-expressing PC-3 prostate tumour xenografts

**DOI:** 10.1186/1471-2407-10-596

**Published:** 2010-10-30

**Authors:** Johanna Tuomela, Tove J Grönroos, Maija P Valta, Jouko Sandholm, Aleksi Schrey, Jani Seppänen, Päivi Marjamäki, Sarita Forsback, Ilpo Kinnunen, Olof Solin, Heikki Minn, Pirkko L Härkönen

**Affiliations:** 1 Institute of Biomedicine, Department of Cell Biology and Anatomy, University of Turku, Turku, Finland; 2 Pharmatest Services Ltd., Turku, Finland; 3 Turku PET Centre, MediCity Preclinical Research Laboratory, University of Turku and Åbo Akademi University, Turku, Finland; 4 Cell Imaging Core, Turku Centre for Biotechnology, University of Turku and Åbo Akademi University, Turku, Finland; 5 Department of Otorhinolaryngology - Head and Neck Surgery, Turku University Hospital, University of Turku, Turku, Finland; 6 Turku PET Centre, Radiopharmaceutical Chemistry Laboratory, University of Turku and Åbo Akademi University, Turku, Finland; 7 Turku PET Centre, Accelerator Laboratory, MediCity Preclinical Research Laboratory, University of Turku and Åbo Akademi University, Turku, Finland; 8 Department of Oncology and Radiotherapy, Turku University Hospital, Turku, Finland; 9 Department of Laboratory Medicine, MAS University Hospital, Lund University, Malmö, Sweden

## Abstract

**Background:**

Prostate tumours are commonly poorly oxygenated which is associated with tumour progression and development of resistance to chemotherapeutic drugs and radiotherapy. Fibroblast growth factor 8b (FGF8b) is a mitogenic and angiogenic factor, which is expressed at an increased level in human prostate tumours and is associated with a poor prognosis. We studied the effect of FGF8b on tumour oxygenation and growth parameters in xenografts in comparison with vascular endothelial growth factor (VEGF)-expressing xenografts, representing another fast growing and angiogenic tumour model.

**Methods:**

Subcutaneous tumours of PC-3 cells transfected with FGF8b, VEGF or empty (mock) vectors were produced and studied for vascularity, cell proliferation, glucose metabolism and oxygenation. Tumours were evaluated by immunohistochemistry (IHC), flow cytometry, use of radiolabelled markers of energy metabolism ([^18^F]FDG) and hypoxia ([^18^F]EF5), and intratumoral polarographic measurements of pO_2_.

**Results:**

Both FGF8b and VEGF tumours grew rapidly in nude mice and showed highly vascularised morphology. Perfusion studies, pO_2 _measurements, [^18^F]EF5 and [^18^F]FDG uptake as well as IHC staining for glucose transport protein (GLUT1) and hypoxia inducible factor (HIF) 1 showed that VEGF xenografts were well-perfused and oxygenised, as expected, whereas FGF8b tumours were as hypoxic as mock tumours. These results suggest that FGF8b-induced tumour capillaries are defective. Nevertheless, the growth rate of hypoxic FGF8b tumours was highly increased, as that of well-oxygenised VEGF tumours, when compared with hypoxic mock tumour controls.

**Conclusion:**

FGF8b is able to induce fast growth in strongly hypoxic tumour microenvironment whereas VEGF-stimulated growth advantage is associated with improved perfusion and oxygenation of prostate tumour xenografts.

## Background

Hypoxia is a common feature of prostate tumours [1]. A low oxygen concentration is known to make tumour cells resistant to radiation therapy and chemotherapy [2]. Furthermore, there is evidence that hypoxia may lead to increasingly malignant behaviour of tumour cells [3,4]. Hypoxia leads to disruption of microenvironmental homeostasis in tumours, where metabolic changes, involving diffusion gradients of oxygen and glucose, develop at microregional level [5]. A common feature of invasive cancers is altered glucose metabolism, including both aerobic and anaerobic glycolysis [6]. Conversion of glucose to lactic acid in the presence of oxygen is known as aerobic glycolysis or the "Warburg effect". The molecular mechanisms leading to upregulated glycolysis in tumours are not well known, but increased glucose uptake and elevated expression of glucose transporters (GLUTs), such as GLUT1, are commonly seen in tumour cells [7]. In prostate cancer, the expression of GLUT1 is also correlated with Gleason score [8]. Members of the GLUT gene family are known to be regulated by hypoxia-inducible transcription factor 1 (HIF1), which is strongly upregulated under hypoxic conditions [9].

Tumour hypoxia is also related with increased angiogenesis which is primarily stimulated by HIF1-induced vascular endothelial growth factor (VEGF) but also by other tumour cell produced cytokines and growth factors including fibroblast growth factors (FGFs) [10,11]. Regardless of neovascularisation, tumours are often poorly oxygenated due to disorganised and leaky vessels [2]. VEGF is a mitogen for vascular endothelial cells but not for other cell types [12,13]. It induces a strong angiogenic response in different *in vivo *models and increases vascular permeability, which is essential to angiogenesis associated with tumour growth and wound healing [14]. In human prostate cancer patients, the expression of VEGF correlates with Gleason score and occurrence of lymph node metastasis [15,16] as well as the poor outcome of radical treatment of localised prostate cancer [17].

The family of FGFs plays an important role in many physiological processes including development [18], wound healing [19], angiogenesis [20], bone formation and osteoblast differentiation [21]. Studies of our own and others on FGF8 have shown that reciprocal FGF/FGF receptor mediated interactions between tumour cells and stromal cells play important roles in prostate cancer progression and angiogenesis [20,22-26]. Fibroblast growth factors 1, 2, 6, 9 and 17 are also expressed at high levels in prostate cancer, where they may function as paracrine and/or autocrine mediators [22,27].

Fibroblast growth factor 8 was originally cloned from conditioned medium of mouse mammary tumour-derived SC-3 cells and was identified as androgen-induced growth factor (AIGF) [28]. Four human FGF8 isoforms, named FGF8a, FGF8b, FGF8e and FGF8f, are formed by alternative splicing [29]. FGF8b has been found to be the most transforming of these isoforms and FGF8b targeted to prostate epithelium causes prostatic intraepithelial neoplasia (PIN) lesions in transgenic mice [30]. FGF8b is also the major isoform expressed in prostate cancer [17,26,31,32]. FGFs mediate their effects by binding to specific tyrosine kinase receptors (FGFR1-4), which all are expressed in prostate cancer [18,33]. The *in vitro *and *in vivo *studies have shown that FGF8b increases growth, invasion, tumorigenesis, angiogenesis and bone metastasis in experimental breast [23,34,35] and prostate cell lines and tumours [20,36-38]. FGF8b is a strongly angiogenic factor, which property has been considered to contribute to increased tumour growth [20,23]. In human prostate cancer, the expression of FGF8b has been shown to predict a poor prognosis [17,32].

We aimed to study whether FGF8b-induced rich neovascularisation is able to influence oxygenation of prostate tumour microenvironment and influence tumour growth by these mechanisms. PC-3 cells were used to create models for prostate cancer expressing VEGF or FGF8b, which both are known to be angiogenic and to be increased in prostate cancer. Nude mice were subcutaneously inoculated with PC-3 cells transfected with FGF8b, VEGF or empty vectors. PC-3/VEGF cells were used as "positive" controls because they were expected to produce well-vascularised and oxygenised tumours. The FGF8b tumours were studied for growth rate, vascularisation, energy metabolism as well as tumour hypoxia and oxygenation, and the findings were compared with those from VEGF and mock tumours, which served as positive and negative controls, respectively.

## Methods

### Cell culture and transfection

The human hormone-resistant prostate cancer cell line PC-3 was obtained from the American Tissue-Type Culture Collection (Rockville, MD, USA). PC-3 cells, which were stably transfected with FGF8b (PC-3/FGF8b) were used as previously described [38]. Stable VEGF transfection was carried out by using the expression vector pcDNA3.1(+) (Invitrogen, CA, USA) containing human VEGF cDNA in an ECORI site (kindly provided by Prof. Kari Alitalo, University of Helsinki, Finland, [39]) and empty pcDNA3.1(+) vector-transfected cells (mock) were used as controls. Neomycin (G418, 500 μg/mL) was added to culture media of transfected PC-3 cells for the selection of clones. At near confluence, the cells were harvested in trypsin/EDTA (Biochrom AG, Germany), washed with culture medium and finally suspended at a concentration of 1 × 10^6^/100 μL in sterile phosphate-buffered saline solution (PBS, Biochrom AG, Germany). The cells were kept on ice until inoculation.

### Northern and Western blot analysis

Total RNA was extracted from PC-3 cells using the guanidinium isothiocyanate method [40]. Northern blotting was performed as previously described [41]. Serum-free DMEM conditioned by FGF8b, VEGF and mock cells was harvested from the cultures as previously described [42]. Heparin-sepharose-bound proteins were extracted by 5-min incubation in Laemmli sample buffer at 95°C and separated by sodium dodecyl sulphate-polyacrylamide gel electrophoresis. After transfer to nitrocellulose membranes (Bio-Rad), proteins were detected by using goat polyclonal anti-FGF8b neutralising antibodies [20] and anti-human VEGF antibodies (both from R&D systems, Minneapolis, MN, USA). Horseradish peroxidase-labelled anti-goat IgG (DAKO, Denmark) was used as a secondary antibody. Protein bands were visualised by using an ECL chemiluminescence detection system (GE, Healthcare Biosciences, Uppsala, Sweden).

### Animals and tumour models

Eight-week-old male athymic nu/nu mice (Harlan, the Netherlands) were maintained under controlled pathogen-free environmental conditions (20-21°C, 30-60% relative humidity and a 12-hour lighting cycle). FGF8b, VEGF and mock cells were inoculated subcutaneously (1 × 10^6 ^cells in 100 μL PBS) into the back of the neck. The animals were monitored daily for clinical signs. Tumour measurements were performed once a week and tumour volume was calculated according to the formula V = (π/6)(d_1 _× d_2_)^3/2 ^[43], where d_1 _and d_2 _are perpendicular tumour diameters. The tumour volume was expressed as mean ± SEM. The animal experiments were carried out according to the European Convention for the Protection of Vertebrate Animals used for Experimental and other Scientific Purposes, plus Statutes 1076/85 § and 1360/90 of The Animal Protection Law in Finland, and EU Directive 86/609. The experiment procedures were reviewed by the local Ethics Committee on Animal Experimentation at the University of Turku and approved by the local Provincial State Office of Western Finland.

### Tumour morphology and immunohistochemical analyses

Morphological evaluation of tumours was determined from frozen sections stained with haematoxylin and eosin (H&E) using standard techniques. Frozen sections (10 μM) were cut, and fixed with ice-cold acetone for 10 minutes at 4°C. Sequential frozen sections were incubated with antibodies against CD31 (BD Biosciences, Pharmingen, CA, USA), Ki67 (Novocastra Laboratories Ltd., Newcastle upon Tyne, UK) and GLUT1 (Alphadiagnostics, TX, USA) o/n at 4°C. HIF1 (BD Biosciences, Pharmingen, CA, USA) antibody was used on formalin-fixed, paraffin-embedded tumour slides o/n at 4°C. The samples were then treated with biotin-labelled rabbit anti-mouse (DAKO Denmark A/S, Glostrup, Denmark or Vectastain CA, USA) secondary antibodies. A mouse-on-mouse kit (Vector Laboratories, Burlingame, CA, USA) was used in the Ki67 antibody staining procedure in order to inhibit non-specific staining of anti-mouse secondary antibodies. Visualisation of the primary antibodies was carried out by using Vectastain ABC reagent and a diaminobenzidine substrate kit (Vector Laboratories, Burlingame, CA, USA), the methodology which is based on the indirect streptavidin-biotin method. The slides were later counterstained with Meyer's haematoxylin. Negative controls (sections of every sample stained without the primary antibody) were used to verify the specificity of staining. Three representative non-overlapping fields inside each tumour were analysed microscopically (Leica, DMRB, Leica Microsystems, Heerburg, Germany) and images were taken with a digital camera (Leica DC 300F, Leica Microsystems, Heerburg, Germany). The lengths of CD31-positive vessels were counted in each tumour by drawing lines following stained vessels and measuring the length of the lines using ImageJ software (ImageJ, 1.37v, Wayne Rasband, National Institutes of Health, USA) [44]. The relative numbers of Ki67- and GLUT1-positive cells were counted in three non-overlapping sections at 500-μm intervals. Altogether, 1.000-3.000 cells/tumour were counted [44]. The results were blind-tested by two independent analysers comparing study versus control samples.

The relative proportion of necrotic tissue was determined in representative H&E-stained tumour slides using the ImageJ software. The evaluation was based on morphological features of the tissue.

The perfusion marker Hoechst 33342 was intravenously injected into tumour-bearing nude mice two minutes before sacrifice in order to study the intratumoral perfusion pattern. Images were acquired from frozen sections (10 μm) using a Zeiss AxioVert 200 M fluorescence microscope (Zeiss GmbH, Jena, Germany).

### Flow cytometry

Three tumours from each group were collected in ice-cold PBS supplemented with pepsin (0.5%, pH 1.4, Merck, Darmstadt, Germany) immediately after sacrifice of the mice. Tumour tissue was homogenised with Ultra-Turrax equipment (Rose Scientific Ltd., Canada) and incubated at 37°C for 1 h. The suspension was collected and filtered prior to analysis. For cell cycle analysis, tumour homogenate was incubated in hypotonic buffer (PBS with 1% Triton X-100 and propidium iodide at 0.05 mg/mL) for 20 min at 4°C. The fractions of cell cycle phases (G0/G1, S and G2/M) were calculated with ModFit cell cycle modelling software (Verity Software House, Inc., Topsham, ME, USA). For cell enumeration, cells were analysed using TrueCount tubes (Becton Dickinson, CA, USA). Briefly, the number of cells in the tube was calculated by comparing a known number of beads in the tube with cell number in a known volume. All flow cytometry was performed using a FACSCalibur (Becton Dickinson, CA, USA) flow cytometer and CellQuestPro software (Becton Dickinson, CA, USA).

### [^18^F]EF5 and [^18^F]FDG uptake in tumours

^18^F-labelled EF5 (2-(2-nitro-1H-imidazol-1-yl)-*N*-(2,2,3,3,3-pentafluoropropyl)-acetamide) was synthesised from 2-(2-nitro-1H-imidazol-1-yl)-*N*-(2,3,3-trifluoroallyl)-acetamide using high specific radioactivity ^18^F-F_2 _as the labelling reagent [45]. The specific radioactivity of [^18^F]EF5, decay corrected to the end of synthesis, exceeded 3.7 GBq/μmol. Radiochemical purity was higher than 98.5% in every production batch. ^18^F-labelled FDG (2-Deoxy-2-fluoro-D-glucose) was synthesised from mannosyl triflate using a nucleophilic method. Radiochemical purity exceeded 95% and specific radioactivity was approximately 74 GBq/μmol at the end of synthesis.

[^18^F]EF5 (FGF8b *n *= 11, VEGF *n *= 12 and mock *n *= 29) and [^18^F]FDG (FGF8b *n *= 6, VEGF *n *= 6 and mock *n *= 5) were intravenously injected (5.6 ± 1.1 and 6.6 ± 1.4 MBq, respectively) into separate FGF8b (tumour weight 408 ± 33 mg), VEGF (tumour weight 506 ± 29 mg), and mock (tumour weight 408 ± 20 mg) tumour-bearing mice, and allowed to distribute 120 minutes before sacrifice. Blood, obtained by cardiac puncture, and tumour tissues were rapidly removed, counted for ^18^F-radioactivity in a well counter (3" × 3" NaI (TI) crystal, Bicron 3MW3/3P, Bicron Inc., Newbury, Ohio, USA) and weighed. The uptake of ^18^F-radioactivity in tissues was calculated as percentage of injected dose per gram of tissue weight, taking into account the background from the counter measurements and the radioactivity decay. Tumour-to-blood uptake ratios (T/B ratios) were then calculated for both [^18^F]EF5 and [^18^F]FDG. Plasma glucose levels were measured (Analox GM9, Analox Instruments Ltd., London, UK) in blood samples (FGF8b *n *= 10, VEGF *n *= 16 and mock *n *= 11).

The intratumoral distribution of [^18^F]EF5 and [^18^F]FDG was studied in FGF8b, VEGF and mock tumour-bearing mice using digital autoradiography. After sacrifice, tumours were removed, rapidly frozen in dry ice/isopentane and cut with a cryomicrotome into 20 μm-thick sections. Tumour sections were then exposed to an imaging plate (Fuji BAS TR2025, Fuji Photo Film Co., Japan) for 3.5-4.5 hours. The spatial distribution of radioactivity from tumour sections was recorded with a phosphoimager (Fujifilm BAS-5000, Fuji Photo Film Co. Ltd., Tokyo, Japan). The dynamic linear range of this system is four decades, and the resolution (i.e. pixel size) of the images is 25 × 25 μm.

The amount of 2-[^18^F]fluoro-2-deoxyglucose-6-phosphate ([^18^F]FDG-6-P), the main metabolite of [^18^F]FDG, was determined in tumour homogenates (FGF8b *n *= 6, VEGF *n *= 4 and mock *n *= 3) from animals injected with [^18^F]FDG by using radioHPLC equipment (Merck Hitachi, Peterlee, UK).

### Oxygen partial pressure (pO_2_) measurement

In order to measure pO_2 _values in tumours, we used sterile, flexible polarographic electrodes (diameter 0.47 mm) of the Clark type (Licox^® ^GMS, Kiel-Mielkendorf, Germany), supplied with a probe-specific microchip allowing automatic calibration. The probe was inserted into the tumour tissue by advancing it in a retrograde manner along the lumen of an insertion needle catheter, which was then removed. Tissue temperature was measured with a needle probe and temperature-adjusted pO_2 _(mmHg) was graphically displayed and stored digitally. The whole length of the oxygen-sensitive part of the probe was at least 2 mm inside the tumour throughout the measurements to prevent contamination from room-air O_2_. The duration of the pO_2 _measurement was sufficient to establish a stable pO_2 _level, which was then registered and stored. Three tumours from each group were measured over a time period of 20 min after a stabilisation period of approximately 5 min. The gluteus muscle of the experimental animal served as a control site after measurements to verify the proper function of the Licox^® ^probe.

### Statistics

All values are presented as mean ± SD unless otherwise stated. Non-parametric one-way ANOVA (Kruskal-Wallis test) with Dunn's *post hoc *test was used for statistical evaluation of CD31, Ki67, GLUT1, HIF1, necrosis, [^18^F]FDG uptake, amount of cells and pO_2 _values. One-way ANOVA with Tukey's *post hoc *test was used for statistical evaluation of plasma glucose levels and [^18^F]EF5 uptake. The above statistical tests were performed using GraphPad Prism version 5.01 for Windows (GraphPad Software, San Diego, CA, USA), based on the distribution of the data (normal or nonparametric) and sample size. A *p*-value less than 0.05 was considered statistically significant.

## Results

### Effects of FGF8b and VEGF on tumour growth and morphology

**Figure 1:**
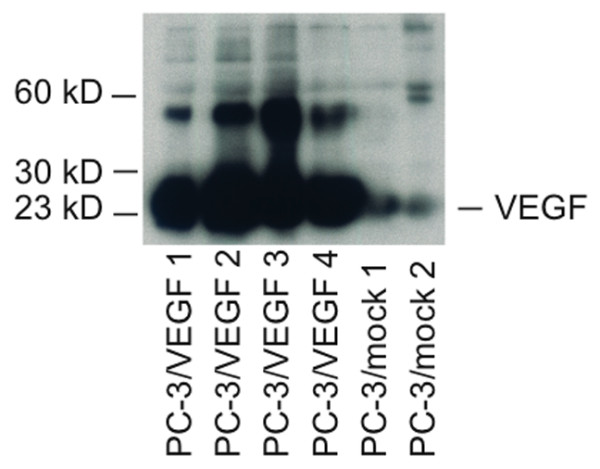
**Expression of VEGF after transfection of PC-3 cells with VEGF or mock expression vectors**. Western blot analysis of conditioned medium of PC-3/VEGF cell clones revealed several clones positive for 23 kD VEGF protein. The larger bands at approximately 60 kD may represent cleaved formes of larger splice variants in these cells. The expression level in mock cells was very low. One PC-3/VEGF clone (PC-3/VEGF3) and one PC-3/mock clone (PC-3/mock2) were selected for *in vivo *studies.

In order to study the roles of FGF8b and VEGF in PC-3 tumours, we used xenografts of PC-3 cells ectopically expressing FGF8b and VEGF. Parental PC-3 cells do not express detectable levels of FGF8b [38] but they express VEGF at a low level (Figure 1). PC-3 cells transfected with FGF8b have been reported previously [38]. To obtain VEGF overexpressing PC-3 tumours, the cells were stably transfected with the expression vector pcDNA3.1(+) containing human VEGF cDNA at an ECORI site. PC-3 cells transfected with empty vectors were used as controls (mock). Expression of VEGF in the isolated clones was confirmed by Western blot analyses (Figure 1) and Northern blot analyses (data not shown). Several clones positive for VEGF were obtained, and one clone (PC-3/VEGF3, later called VEGF) was selected for *in vivo *studies.

**Figure 2:**
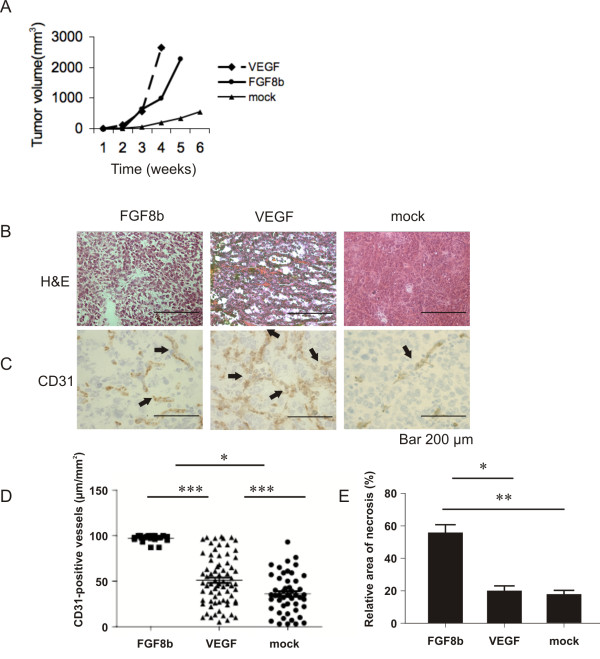
**Growth and morphology of FGF8b, VEGF and mock tumours**. A, Growth of subcutaneous FGF8b, VEGF and mock tumours (*n *= 30, *n *= 22 and *n *= 14, respectively). Tumour diameter in 2 perpendicular dimensions was measured once a week and tumour volume was calculated according to the formula V = (π/6)(d_1 _× d_2_)^3/2 ^and presented as a function of time (mean ± SEM). Differences in tumour volumes between the groups were significant at all time points between 2 and 4 weeks (*p *< 0.001). B, H&E staining of representative FGF8b, VEGF and mock tumours (Bar 200 μm). C-D, The density of CD31-positive blood capillaries (μm/mm^2^) was counted in a blinded manner from 3 fields of the FGF8b, VEGF and mock tumours (51 ± 27 μm/mm^2^, *n *= 18, 97 ± 4 μm/mm^2^, *n *= 72, and 36 ± 21 μm/mm^2^, *n *= 49, respectively), p* < 0.05, p*** < 0.001 (Bar 200 μm). E, The relative area of necrosis was counted in a blinded manner from 3 fields in FGF8b (*n *= 6), VEGF (*n *= 6) and mock (*n *= 6) tumours, p* < 0.05, p** < 0.01.

Tumour growth associated with PC-3/FGF8b, PC-3/VEGF and PC-3/mock cells was studied by inoculating them s.c. in the back of the necks of nude mice. The average growth (mean ± SEM) of FGF8b, VEGF and mock tumours is shown in Figure 2A. Mock-transfected cells produced only small tumours (749 ± 19 mm^3^), whereas FGF8b and VEGF tumours grew larger (3436 ± 41 mm^3 ^and 4036 ± 51 mm^3^, respectively) during the 6 and 4-week study periods, respectively (*p *< 0.001).

Both FGF8b and VEGF tumours showed strong angiogenic morphology. They were reddish in colour and many of the FGF8b tumours were fragile and haemorrhagic. The morphology of mock tumours was solid, homogeneous and similar to parental PC-3 tumours. Morphological examination of the H&E-stained sections showed a rich capillary network and large sinusoid-like vessels, especially in VEGF tumours (Figure 2B). Capillaries in FGF8b tumours looked more aberrant, containing large cisternal spaces filled with blood (Figure 2B). The number of capillaries was modest in mock tumours (Figure 2B). Vascularisation was verified using immunohistochemical staining for the endothelial marker CD31 and this procedure confirmed the differences in blood capillary density between these three different tumour types (Figure 2C). Significantly increased density of capillaries (mean ± SEM, *p *< 0.001) was seen in both FGF8b (51 ± 27 μm/mm^2^) and VEGF (97 ± 4.51 μm/mm^2^) tumours compared with that found in the mock tumours (36 ± 21 μm/mm^2^, Figure 2C-D).

The relative area of necrosis was measured in H&E-stained tumour sections. The data demonstrated that the relative area of necrosis was larger in the FGF8b tumours than in the VEGF (*p *< 0.05) or mock (*p *< 0.01) tumours (Figure 2E).

### Cell cycle analysis and proliferation

**Figure 3:**
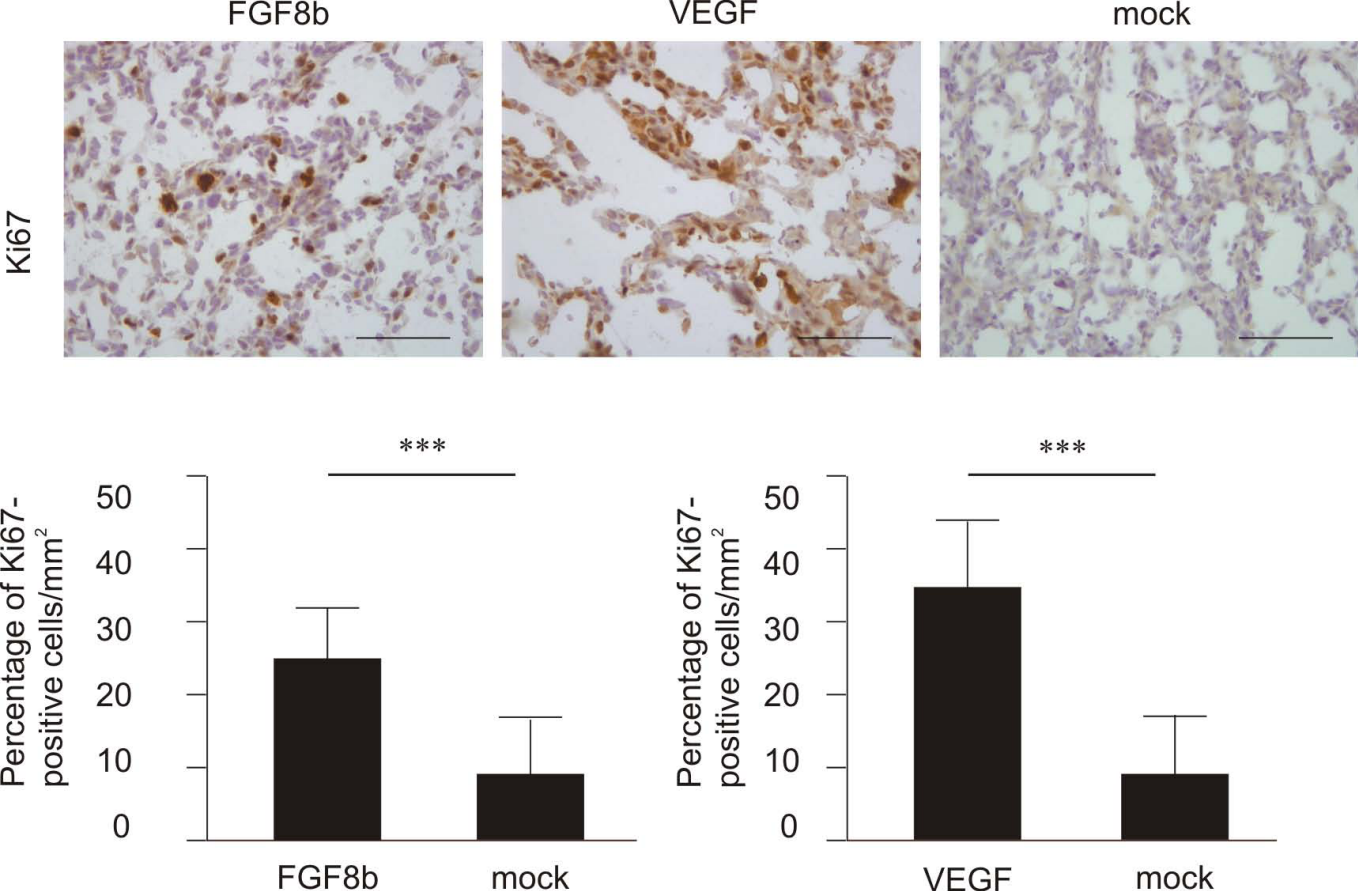
**The effect of ectopic FGF8b and VEGF on proliferation**. Ki67 immunostaining of FGF8b (*n *= 40), VEGF (*n *= 5) and mock (*n *= 23) tumours (Bar 200 μm). The relative number of Ki67-positive cells in tumours was counted in a blinded manner in 3 fields per tumour and the results were expressed as percentage of positive cells per mm^2^. There were significantly more proliferative cells (*p *< 0.001) in both FGF8b and VEGF tumours compared with mock tumours.

Cell cycle phases of FGF8b, VEGF and mock tumours were determined using flow cytometry. These analyses revealed a higher number of S-phase cells in both FGF8b (21%) and VEGF (16%) tumours compared with mock tumours (12%) (data not shown). Immunohistochemical staining of tumour sections for Ki67 confirmed these findings (Figure 3), showing a significantly higher proliferation rate of FGF8b and VEGF tumours compared with that of mock tumours (*p *< 0.001). The percentage of Ki67-positive cells per mm^2 ^was 26 ± 11, 33 ± 10 and 10 ± 7 in FGF8b, VEGF and mock tumours, respectively (Figure 3).

### Perfusion and oxygenation status of tumours

**Figure 4:**
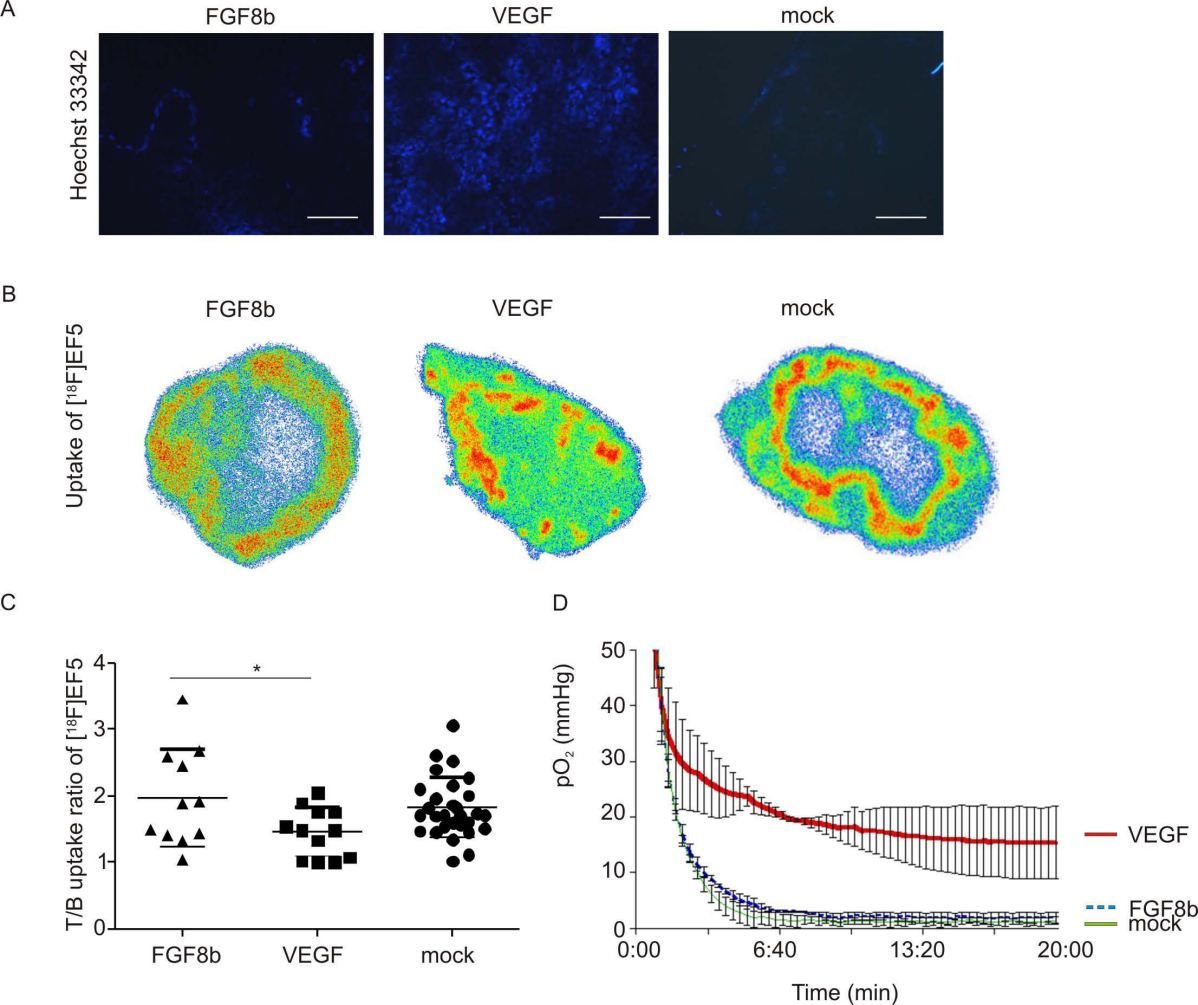
**Perfusion and oxygenation status of tumours**. A, Labelling with the perfusion marker Hoechst 33342 showed that the flow in blood vessels was better in the VEGF tumours compared with the FGF8b and mock tumours, where Hoechst 33342 labelling was detected only in the tumour periphery (Bar 200 μm). Examples of the intratumoral distribution of [^18^F]EF5 in FGF8b, VEGF and mock tumour sections are shown in B. FGF8b and mock tumours showed mainly peripherally located uptake of [^18^F]EF5, whereas uptake into VEGF tumours was more uniform throughout the tumours. Uptake intensity is not comparable between these images, since they were not corrected for injected dose or cross-calibrated between separate studies. C, The tumour-to-blood (T/B) uptake ratio of [^18^F]EF5 (T/B ratio) in FGF8b (*n *= 11), VEGF (*n *= 12) and mock (*n *= 29) tumours is expressed as mean ± SD. Accumulation of [^18^F]EF5 was significantly lower (*p *< 0.05) in VEGF tumours compared with mock tumours, whereas no significant difference in T/B ratio was seen between FGF8b and mock tumours. Partial pressures of oxygen (pO_2_) in VEGF (*n *= 3), FGF8b (*n *= 3) and mock (*n *= 3) tumours are shown in D. The mean values of pO_2 _measurements are shown as a curve. VEGF tumours were relatively well oxygenated in comparison with FGF8b and mock tumours.

The labelling intensity of the fluorescent perfusion marker Hoechst 33342 displayed distinct differences in perfusion patterns among the tumours. Labelling intensity was very weak in FGF8b tumours, indicating poor perfusion. As expected, the intensity was strongest in the VEGF tumours (Figure 4A), where signals were detected in both central and peripheral areas, suggesting that VEGF tumours are well perfused. Labelling intensity was modest in the mock tumours.

The nitroimidazole family compound EF5 forms covalent bonds with cellular macromolecules under hypoxic conditions, and therefore tends to accumulate at hypoxic sites [45,46]. The intratumoral distribution of [^18^F]EF5 determined by digital autoradiography in tumour sections showed a wide variation in the hotspot areas inside tumours (Figure 4B). The uptake pattern was very similar with that we have earlier detected and reported in regard to other hypoxia tracers [47]. It was not possible to ascertain clear divisions based on the intratumoral uptake pattern of [^18^F]EF5 according to tumour size, or the expression levels of the studied growth factors. In general, however, some distinguishable trends were detected. Variation in the intratumoral distribution of [^18^F]EF5 in smaller tumours (~200 mg) was similar (data not shown) in all groups. Larger tumours (~500 mg) tend to show more uniformly distributed hotspots of [^18^F]EF5 uptake in VEGF-expressing tumours, whereas uptake of [^18^F]EF5 was primary located in peripheral parts of FGF8b- and mock-tumours (Figure 4B). The accumulation of [^18^F]EF5, in whole tumours was measured after a distribution time of 120 min. As shown in Figure 4C, a significantly lower T/B uptake ratio (*p *< 0.05) of [^18^F]EF5 was found in VEGF tumours (1.45 ± 0.37) compared with that measured in mock tumours (1.81 ± 0.45). The mean T/B uptake ratio (1.97 ± 0.73) of [^18^F]EF5 in FGF8b tumours was not significantly different compared to VEGF or mock tumours (Figure 4C).

In order to confirm these findings, we measured the intratumoral partial pressure of oxygen (pO_2_) using a Licox^® ^Revoxode CC1.2 polarographic probe (Figure 4D). Significantly lower (*p *< 0.001) mean pO_2 _values were measured in both FGF8b and mock tumours (2.3 ± 0.6 mmHg and 1.3 ± 0.3 mmHg, respectively) compared with that in VEGF tumours (17.4 ± 2.2 mmHg). The pO_2 _value of the gluteus muscle, measured as a control, was 45 mmHg (data not shown).

**Figure 5:**
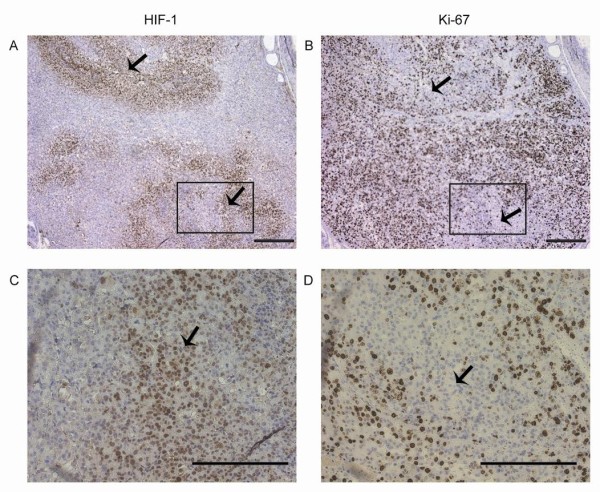
**Relationship between tumour hypoxia and proliferation**. Localisation of areas positively stained for HIF1α and Ki67 was visually detected from microscope images in FGF8b and mock tumours. The arrows show positive HIF1α staining and negative Ki67 staining in A and B, and C and D. C and D are captions from the indicated areas in A and B, respectively (Bar 200 μm).

In addition to autoradiography, hypoxic clusters in FGF8b and mock tumors were also detected by immunostaining of hypoxia-inducible factor (HIF1α) [48,49]. As shown in Figure 5, the areas staining strongly for HIF1α showed little immunopositivity for Ki67 whereas the areas with Ki67 positive cells did not markedly stain for HIF1.

### Metabolic activity of tumours

**Figure 6:**
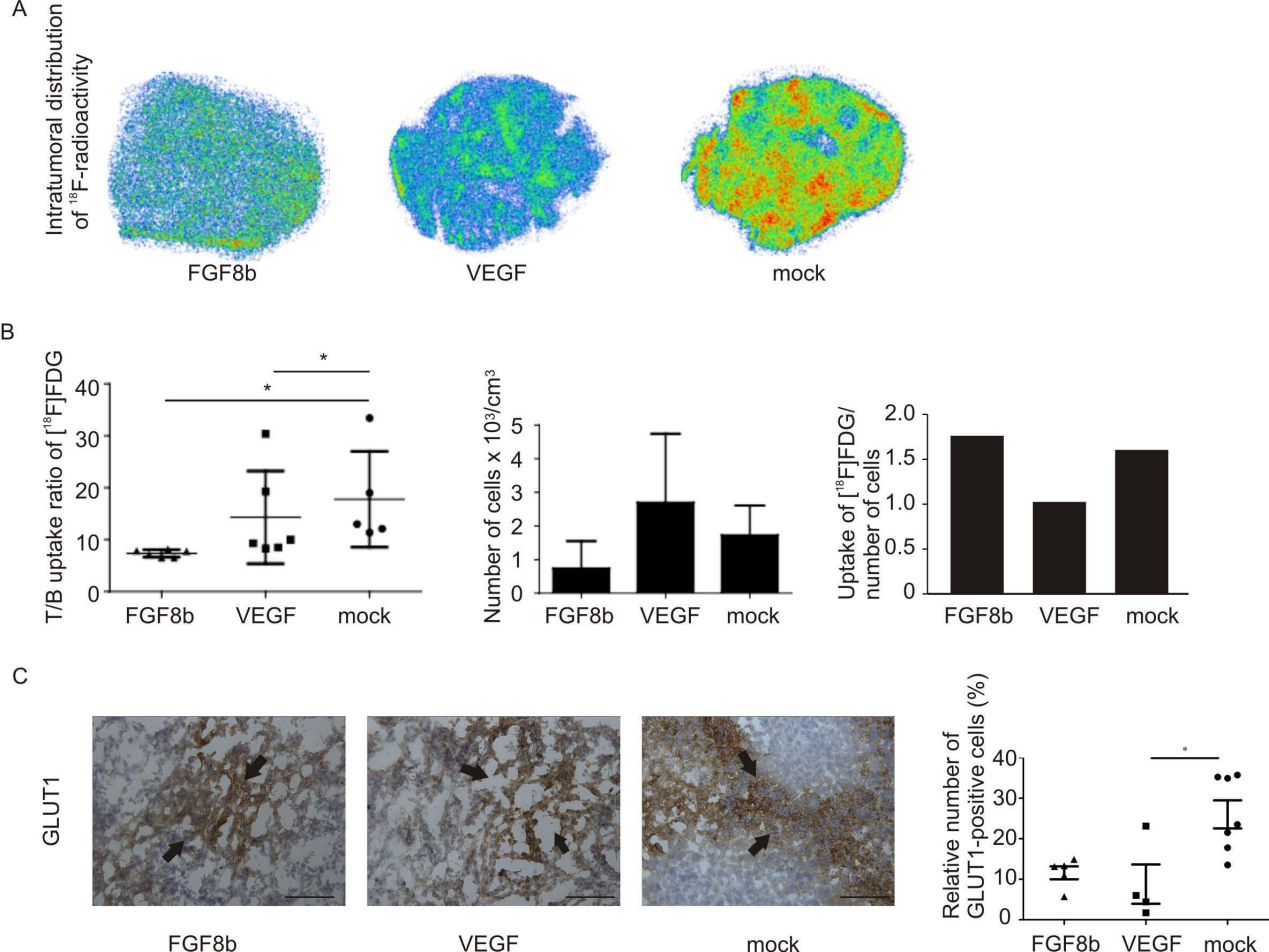
**Glucose metabolism**. A, Intratumoral distribution of [^18^F]FDG-derived radioactivity in tumour sections (FGF8b *n *= 6, VEGF *n *= 6 and mock *n *= 5). Uptake intensity is not comparable between these images, since they were not corrected for injected dose or cross-calibrated between separate studies. B, Tumour-to-blood (T/B) uptake ratio of [^18^F]FDG (T/B ratio) in FGF8b (*n *= 6), VEGF (*n *= 6) and mock (*n *= 5) tumours is expressed as mean ± SD. The accumulation of [^18^F]FDG was significantly lower (*p *< 0.05) in FGF8b tumours compared with VEGF and mock tumours. Number of cells/cm^3 ^was determined using TrueCount tubes and flow cytometry. On the right, we show relative uptake of [^18^F]FDG after balancing the uptake against cell number. C, Immunostaining of the glucose transporter GLUT1 showed decreased staining in VEGF tumours compared with mock and FGF8b tumours (FGF8b *n *= 5, VEGF *n *= 4 and mock *n *= 7 tumours) (*p *< 0.05 VEGF *vs*. mock, Bar 200 μm). Analysis was carried out in a blinded manner in three representative non-overlapping fields of the tumours, and the data presented as mean ± SD.

In order to evaluate the metabolic activity of FGF8b- and VEGF-expressing tumours, we measured the uptake of [^18^F]FDG-derived radioactivity. The intratumoral distribution of [^18^F]FDG determined by digital autoradiography showed uniformly distributed uptake in tumours (Figure 6A). We found a significantly lower (*p *< 0.05) T/B uptake ratio of [^18^F]FDG in FGF8b tumours (7.4 ± 0.7) compared with that seen in VEGF and mock tumours (14.3 ± 8.9 and 17.8 ± 9.3, respectively), as shown in Figure 6B. The intracellular [^18^F]FDG-6-P (the main metabolite of [^18^F]FDG, which is trapped inside the cell) was detected by radioHPLC. The amount did not differ between the tumour groups (data not shown). The mean plasma glucose level varied from 7.9 to 10.6 mmol/L and was not significantly different in the three groups of tumour-bearing mice.

Since morphological studies indicated a lower number of cells in FGF8b tumours, which would affect the relative level of [^18^F]FDG uptake, we evaluated cellular density in the different tumour models by flow cytometry. Enumeration analysis showed that indeed FGF8b tumours contained fewer cells/cm^3 ^than VEGF and mock tumours (Figure 6B). A similar result was obtained by counting nuclei per field in H&E-stained sections (data not shown). When the [^18^F]FDG uptake (percentage of injected dose per gram tissue) was normalised to cell number, the differences between FGF8b and mock tumours disappeared, and according to the normalised data, the uptake was smallest in VEGF tumours (Figure 6B). Immunohistochemical staining of GLUT1 (Figure 6C) correlated with the uptake of [^18^F]FDG, showing a trend towards a lower expression pattern of GLUT1 in VEGF tumours compared to mock tumours (*p *< 0.05).

## Discussion

Tumour vasculature provides necessary oxygen and nutrients for tumour growth, but it also plays a major role in metastatic spread. In prostate cancer, VEGF-mediated angiogenesis has proved to be important for tumour growth and metastasis [10,50]. VEGF receptors are overexpressed in prostate cancer and plasma levels of VEGF are raised in metastatic disease compared with localised disease or healthy controls. Alterations in the vascular supply influence the tumour microenvironmental conditions, leading to changes in blood flow, pH, glucose metabolism and oxygenation, which all may have significant effects on the responses of tumours to therapy [5]. Prostate tumours are typically very hypoxic. Hypoxia is known to induce a more aggressive phenotype, which increases metastatic potential, promotes tumour progression and limits the effectiveness of radiation therapy [2,51]. Tumour cells generally show increased glycolysis, even under aerobic conditions, and a rough correlation between the degree of malignancy and glycolytic rate has long been recognised [52].

The purpose of this study was to evaluate the role of angiogenesis and tumour oxygenation in stimulation of growth of PC-3 prostate xenografts representing commonly hypoxic prostate cancer. Morphologic evaluation of PC-3 tumours overexpressing FGF8b, as well as those over-expressing VEGF, showed angiogenic morphology overall, compared with mock tumours. According to the results of our previous studies, FGF8b may be a key regulator of prostate cancer angiogenesis, supporting tumour growth [20]. We have previously shown that FGF8b increases angiogenic capacity in breast cancer cells and increases vessel sprouting in a chorion allantoic membrane assay [34,35]. In the present study, immunostaining with the endothelial marker CD31 confirmed that both FGF8b- and VEGF-expressing tumours had rich networks of capillaries, while in mock tumours the capillary network was sparse and individual capillaries were smaller. However, the capillary networks in angiogenic FGF8b- and VEGF-expressing tumours were not similar. Both tumour models showed large numbers of capillaries, but the capillary network was more distorted and non-continuous in FGF8b tumours. Areas of cisternal spaces filled with blood were also detected in FGF8b tumours. The accelerated growth rate of both FGF8b and VEGF tumours indicated an increased vessel capacity to support and contribute to tumour growth. FGF8 has also been shown to stimulate the rate of proliferation of PC-3 cells *in vitro *[20,36], while VEGF is a mitogenic factor affecting endothelial cells only. In the present study, the number of Ki67-positive cancer cells was significantly increased in both FGF8b and VEGF tumours (Figure 3). However, morphological examination of the tumours revealed significantly widened areas of necrosis in FGF8b tumours compared with the others. It has been previously shown that FGFs may not be able to induce mature and fully functional capillaries without synergistic actions of other growth factors [53]. These findings made us question whether the capillaries in FGF8b tumours are able to function properly.

The performance of blood capillaries was studied by means of the perfusion marker Hoechst 33342, which revealed relatively low perfusion in mock tumours. Functional capillaries were seen only in the periphery of the tumours and they were sparse in central parts (Figure 4A). Despite the rich capillary network in FGF8b tumours, very low perfusion was detected, indicating that the rapid growth in comparison with mock tumours was caused by factors other than increased blood flow. It seems that the number of functional capillaries in FGF8b tumours is modest. The vessel-like structures could be a consequence of angiogenic mimicry, and these structures are not able to maintain proper oxygenation and support intratumoral cells. On the other hand, the VEGF tumours seemed to have an adequate number of functional capillaries. VEGF tumours were properly perfused, not only peripherally but also in central areas. Accordingly, the area of necrotic tissue in VEGF tumours was negligible.

Because of abnormal vasculature, hypoxia is very common in solid tumours. Since morphological evaluation and perfusion studies of the FGF8b tumours indicated that these vessels were not functioning normally, we decided to evaluate the proportion of hypoxic cells in the tumours. We found a significantly reduced T/B uptake ratio of the hypoxia tracer [^18^F]EF5 in VEGF tumour-bearing animals, indicating that these tumours are indeed more perfused and oxygenated than the others (Figure 4). The intratumoral distribution of [^18^F]EF5 was studied autoradiographically. Uptake of [^18^F]EF5 was generally in accordance with the results of the perfusion study, showing a relatively high level of [^18^F]EF5 uptake in large peripheral regions of FGF8b and mock tumours, whereas VEGF tumours showed a more uniform uptake, seen as smaller clusters throughout the tumour.

The partial pressure of oxygen (pO_2_) seen in healthy tissues varies from 95 mmHg in arterial blood to a mean value of about 40 mmHg in tissues. Usually hypoxia is defined as an O_2 _concentration below 10 mmHg and pO_2 _values less than 5 mmHg are generally considered as severe hypoxia [2]. According to our oxygen measurements, both the FGF8b and mock tumours were hypoxic, showing pO_2 _values of 2.3 ± 0.6 mmHg and 1.3 ± 0.3 mmHg, respectively, whereas VEGF tumours were well oxygenated (17.4 ± 2.2 mmHg). Even if there were intratumoral necrotic areas, the difference seemed to be significant. Importantly, tumour hypoxia is generally considered as a poor prognostic factor [54]. In order to evaluate the hypoxia status of FGF8b and mock tumours further, we immunostained tumours against HIF1α, which mediates acute molecular responses to hypoxia [49]. The HIF1α-positive cells in FGF8b and mock tumours were seen as hypoxic clusters. When these clusters were visually evaluated from microscope images and compared to the expression of Ki67, we found a negatively related expression pattern.

In order to evaluate the metabolic activity of the tumours, we used [^18^F]FDG, a glucose analogue, that is trapped inside metabolically active cells in the form of [^18^F]FDG-6-P [55]. As expected, relatively high uptake of [^18^F]FDG was seen in all three tumour models. Surprisingly, the lowest T/B ratio was seen in FGF8b tumours. This can be partly explained by the lower cell density and increased intracellular space seen in the FGF8b tumours compared with the mock and VEGF tumours (Figure 2B and 6C). When [^18^F]FDG uptake was balanced against cell number, the level of uptake in FGF8b tumours was similar to that in the mock tumours. Expression of the glucose transporter GLUT1 was also increased in FGF8b and mock tumours, which indicates that hypoxic tumours compensate for their hypoxia by increased glucose consumption [6]. Hypoxic cells often show enhanced glycolysis to maintain production of energy in the form of ATP without requiring O_2 _[6]. The results of several studies have shown a relationship between [^18^F]FDG accumulation and GLUT1 expression in cancer [56]. The relationship between hypoxia and glucose metabolism might, however, be more complicated than is generally believed [57]. In a study by Rajendran *et al. *the authors found a wide variation in the relationship between hypoxia and energy metabolism in patients with different forms of cancer [58]. Acute hypoxia can stimulate anaerobic glycolysis in normal tissues and also in some tumours, but it is not a prime cause of glycolysis, which is the preferred means of energy metabolism in many tumours under aerobic conditions as well [6]. Glucose metabolism is also influenced by factors other than hypoxia, such as the number of tumour-infiltrating immunoreactive cells. Cellular proliferation might be reduced in chronically hypoxic regions, whereas the proliferation rate can be elevated in non-hypoxic tumour areas. This kind of cellular stress often activates other metabolic pathways as well, such as fatty acid synthetase (FAS) pathways [59]. Enhanced glycerolipid/free fatty acid cycling, on the other hand, favours tumour cell growth in environment poor in nutrients [60]. In addition, the expression of mitogenic FGF8b itself increases tumour growth. In clinical practice, high metabolic activity and hypoxia are considered to be characteristics of aggressive tumours [58]. Increased expression of FGF8b is known to predict poor clinical outcome of the patient [17,32] but it remains to be studied whether hypoxic tumour microenvironment is associated with or contributes to FGF8b-driven tumour progression.

The limitation of our experimental study is that subcutaneous tumours instead of orthotopic tumours were used, which means heterotopic tumour microenvironment for tumour cells. The PC-3 control tumours formed were, however, hypoxic as clinical prostate tumours are. Another limitation is that the expression of FGF8b in PC-3 cells was not endogenous but produced by transfection of the ectopic gene which did not allow studies on the possible effects of silencing the gene on tumour microenvironment. Unfortunately, no prostate cancer cell line expressing FGF8 is available. Silencing endogenous VEGF would also add to the results of the role of VEGF in tumour oxygenation and tumour growth in our model. Additionally, studies of correlations of VEGF, FGF8 and HIF1 with each other and characteristics of clinical tumour samples and outcome of prostate cancer patients would clarify the role and significance of FGF8 in regulation of prostate cancer progression.

Our study revealed that our prostate tumour models have different profiles as regards hypoxia, perfusion and metabolism. The relationship between hypoxia and blood flow seems to be complex, and our results thus support the theory of variable oxygenation status coupled to a highly vascular morphology but differential numbers of functional vessels. Our results also support earlier evidence that hypoxia and accelerated glycolysis are common but independent phenomena in a malignant tumour phenotype. Our results are in line with those of studies on human prostate cancer, where the expression of FGF-8, VEGF and clinicopathological findings correlate with each other [61]. FGF8b could thus, alone or combined with other markers, be used as a prognostic indicator in cases of prostate cancer. It remains to be studied whether FGF8 expression is able to contribute to development of resistance to radiation and cytotoxic therapies of prostate cancer.

## Conclusions

Our results suggest that while VEGF-increased prostate tumour growth is associated with and most probably caused by increased density and performance of the vascular network. Elevated tumour growth in FGF8b expressing xenografts is based on stimulated proliferative activity and increased hypoxia tolerance, which may promote prostate tumour progression to a more aggressive phenotype associated with resistance to radiation and cytotoxic therapies.

## Abbreviations

AIGF: androgen-induced growth factor; DMEM: Dulbecco's modified Eagle's medium; ECL: enhanced chemiluminescence; EDTA: ethylenediaminetetra-acetic acid; EF5: (2-(2-nitro-1H-imidazol-1-yl)-*N*-(2,2,3,3,3-pentafluoropropyl)-acetamide); FAS: fatty acid synthetase; FDG: fluoro-2-deoxyglucose; FDG-6-P: fluoro-2-deoxyglucose-6-phosphate; FGF: fibroblast growth factor; FGFR: fibroblast growth factor receptor; GLUT: glucose transport protein; H&E: hematoxylin-eosine; HPLC: high performance liquid chromatography; iFBS: heat-inactivated fetal bovine serum; IHC: immunohistochemistry; PBS: phosphate-buffered saline; PIN: prostatic intraepithelial neoplasia; pO2: partial oxygen pressure; s.c.: subcutaneous; T/B: tumour to blood ratio; VEGF: vascular endothelial growth factor.

## Competing interests

The authors declare that they have no competing interests.

## Authors' contributions

JT carried out the cell cultures, RNA isolation, Northern Blot analysis, Western Blot analysis, analysis of morphology and morphometry. Transfection of the PC-3 cell was done by MV and JSe. Tumour experiments were done by JT and TG. Flow cytometric analysis was made by JSa. AS and IK helped with partial oxygen measurement. PM helped with processing the tumours. SF made the radiochemical synthesis of tracers. TG made the analysis of uptake of tracers and statistical analysis. PH, HM and OS participated in the design of the study. JT wrote the first version of the manuscript and all authors helped to process it. All authors have read and approved the final manuscript. PH gave final approval for the manuscript to be submitted.

## Pre-publication history

The pre-publication history for this paper can be accessed here:

http://www.biomedcentral.com/1471-2407/10/596/prepub
